# Zika virus like particles elicit protective antibodies in mice

**DOI:** 10.1371/journal.pntd.0006210

**Published:** 2018-02-05

**Authors:** Mauricio A. Salvo, Brock Kingstad-Bakke, Cristhian Salas-Quinchucua, Erwin Camacho, Jorge E. Osorio

**Affiliations:** Department of Pathobiological Sciences, School of Veterinary Medicine, University of Wisconsin, Madison, WI, United States of America; Oregon Health and Science University, UNITED STATES

## Abstract

Mosquito-borne Zika virus (ZIKV) typically causes a mild and self-limiting illness known as Zika fever. Since its recent emergence in 2014 in the American continent, ZIKV infection during pregnancy has been closely associated with a wide range of congenital abnormalities. To date, no vaccines or antivirals are publicly available. We developed Zika virus-like particles (VLPs) and evaluated their immunogenicity and protective efficacy in mouse models. ZIKV VLPs (ZIKVLPs) formulated with alum were injected into 6-8-week-old interferon deficient AG129 mice as well as wild type BALB/c mice. Control mice received PBS/alum. Animals were challenged with 200 PFU (>1000 AG129 LD50s) of ZIKV strain H/PF/2013. All vaccinated mice survived with no morbidity or weight loss while control animals either died at 9 days post challenge (AG129) or had increased viremia (BALB/c). Neutralizing antibodies were observed in all ZIKVLP vaccinated mice. The role of neutralizing antibodies in protecting mice was demonstrated by passive transfer. Our findings demonstrate the protective efficacy of the ZIKVLP vaccine and highlight the important role that neutralizing antibodies play in protection against ZIKV infection.

## Author summary

Mosquito-borne Zika virus (ZIKV) typically causes a mild and self-limiting illness known as Zika fever. During the recent outbreak in South America, ZIKV infection during pregnancy was associated with severe congenital abnormalities. We developed a vaccine against ZIKV utilizing virus-like particles (VLPs). VLPs are structurally similar to viruses, but are not infectious. We injected mutant mice capable of succumbing to ZIKV infection with these VLPs. Mice vaccinated with VLPs survived infection, while negative control mice died. These studies are important because ZIKVLP based vaccines could be tested in humans as a prophylactic candidate with minimal safety concerns able to protect unborn babies whose mothers become infected with Zika virus during pregnancy.

## Introduction

Zika virus (ZIKV; *Flaviviridae*, *Flavivirus*) is an emerging arbovirus, transmitted by *Aedes* mosquitoes [1]. ZIKV has a positive-sense, single-stranded RNA genome, approximately 11 kilobases in length that encodes three structural proteins: the capsid (C), premembrane/membrane (prM), and envelope (E), and seven non-structural proteins (NS1, NS2A, NS2B, NS3, NS4A, 2K, NS4B, and NS5). Based on a genetic study using nucleotide sequences derived from the NS5 gene, there are three ZIKV lineages: East African, West African, and Asian [2] [3]. ZIKV emerged out of Africa and previously caused outbreaks of febrile disease in the Yap islands of the Federated states of Micronesia [4], French Polynesia [5], and Oceania. Currently, nearly all Latin American countries have experienced local transmission ZIKV [6], and there are now reports of mosquito transmitted ZIKV in North America. The current outbreak in the Americas is cause for great concern, because of the fast and uncontrolled autochthonous spread, and unique ability among ZIKV to transmit through sexual contact [7]. Clinically, infection with ZIKV resembles dengue fever and several other arboviral diseases [8], but infection during pregnancy has been linked to fetal neurological syndromes and congenital malformation [9]. Alarmingly, the rate of microcephaly (small head, reduced brain size, impaired neurocognitive development) in infants born has increased significantly (20-fold in 2015) in areas with high ZIKV incidence in South America [10] [11, 12], and possibly the US [13]. ZIKV infection in adults has also been strongly linked to Guillain-Barré syndrome (GBS) and meningoencephalitis [14]. In February 2016, the World Health Organization declared the Zika virus an international public health emergency, prompted by its link to microcephaly.

To date, there are no ZIKV licensed vaccines or antiviral therapies available for treatment. ZIKV envelope glycoproteins have been identified as good candidates for vaccine development, as these have been correlated to the induction of potent ZIKV-specific neutralizing antibodies[15, 16]. Several approaches are currently being explored to develop a ZIKV vaccine, including inactivated, recombinant live-attenuated viruses, protein subunit vaccines, virus-like particles (VLPs), RNA, and DNA vaccines[17–21]. A VLP vaccine approach against ZIKV would eliminate concerns of live attenuated vaccines and insufficient inactivation of killed vaccines for pregnant women and other populations at high risk of suffering the devastating effects of ZIKV infections.

In recent years, recombinant VLP-based vaccine strategies have been frequently used for vaccine design. VLPs are empty, non-infectious viral capsids that contain viral proteins. VLPs are known to be highly immunogenic and elicit higher titer neutralizing antibody responses than subunit vaccines based on individual proteins [22]. Such VLPs present viral spikes and other surface components that display linear or conformational epitopes in a repetitive array that effectively results in recognition by B-cells [23]. This recognition leads to B cell signaling and MHC class II up-regulation that facilitates the generation of high titer specific antibodies. VLPs from viruses, including hepatitis B virus, West Nile virus and chikungunya virus, elicit high titer neutralizing antibody responses that contribute to protective immunity in preclinical animal models and in humans [24–26].

Here, we report the development of a ZIKVLP vaccine and its evaluation in susceptible interferon AG129 mice, as well a wild-type BALB/c model. Our findings demonstrate the protective efficacy of the ZIKVLP vaccine and highlight the important role that neutralizing antibodies play in protection against ZIKV infection.

## Materials and methods

### Cells and viruses

African Green Monkey kidney cells (Vero) and Human embryonic kidney 293 (HEK293) were obtained from ATCC (ATCC; Manassas, VA, USA) and grown in Dulbecco’s modified Eagle medium (DMEM) supplemented with 10% fetal bovine serum (FBS; Hyclone, Logan, UT), 2 mM L-glutamine, 1.5 g/l sodium bicarbonate, 100 U/ml of penicillin, 100 μg/ml of streptomycin, and incubated at 37°C in 5% CO2. ZIKV strain H/PF/2013 (GenBank:KJ776791), was obtained from Xavier de Lamballerie (European Virus Archive, Marseille France). Virus stocks were prepared by inoculation onto a confluent monolayer of Vero cells.

### Animals

Mice of the 129/Sv background deficient in alpha/beta interferon alpha/beta/gamma (IFN-α/β/IFN-γ) receptors (AG129 mice) were obtained from B&K Universal Limited (Hull, England) and were bred in the pathogen-free animal facilities of the University of Wisconsin-Madison School of Veterinary Medicine. 5-week-old BALB/c mice (The Jackson Laboratory, Maine, USA) were used for wild-type vaccination studies. Groups of mixed sex mice were used for all experiments.

### Ethics statement

This study was carried out in strict accordance with recommendations set forth in the National Institutes of Health Guide for the Care and Use of Laboratory Animals. All animals and animal facilities were under the control of the School of Veterinary Medicine with oversight from the University of Wisconsin Research Animal Resource Center. The protocol was approved by the University of Wisconsin Animal Care and Use Committee (Approval #V01327).

### Production and purification of ZIKV VLPs

**Fig 1:**
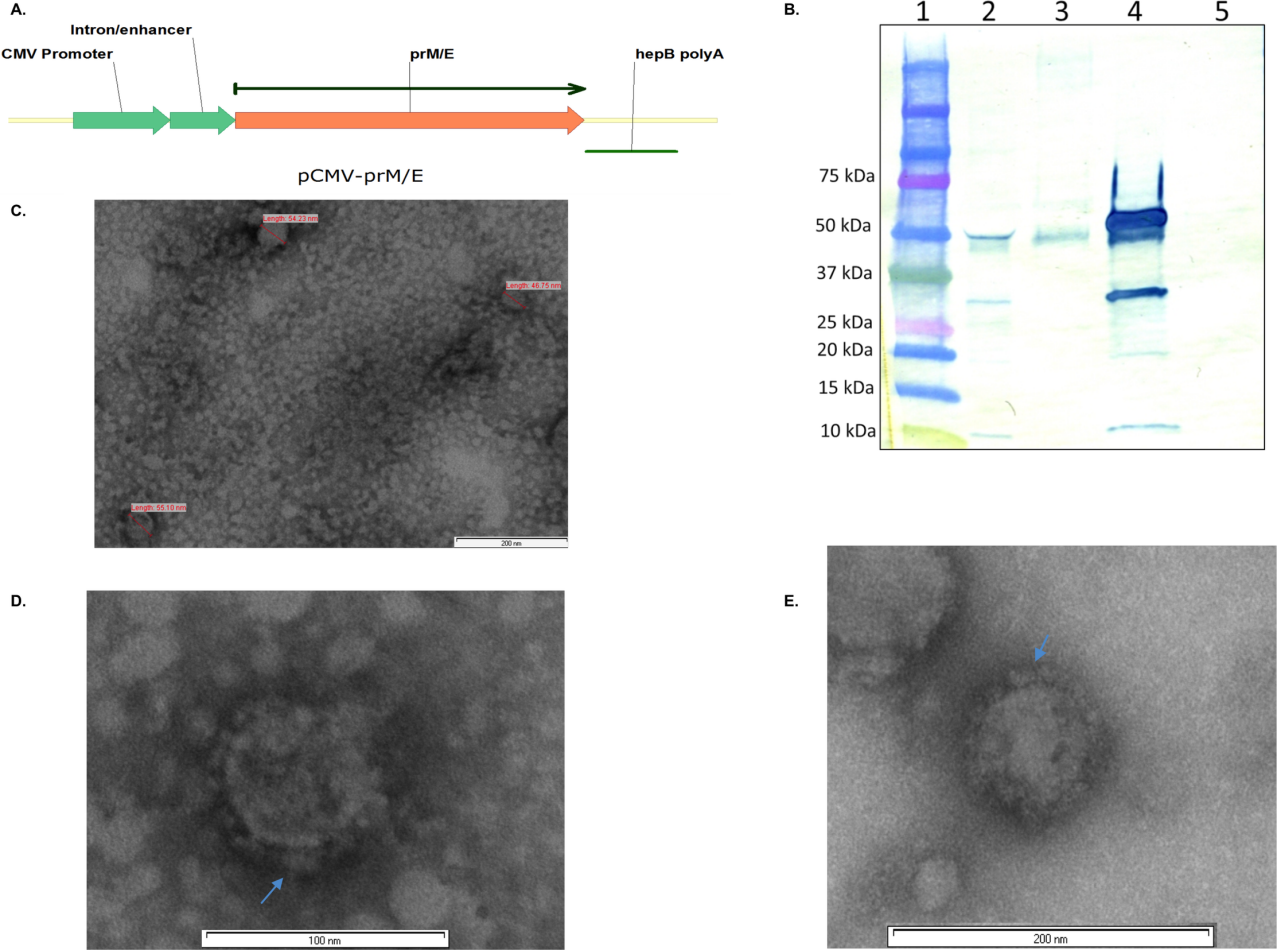
*In vitro* characterization of Zika virus like particles. 1A: Schematic of pCMV-prM/E expression cassette. 1B: Western blot analysis of Zika virus like particles. Lanes are, 1) Bio-rad precision plus kaleidoscope protein standards. 2): pCMV-prM/E transfection pre sucrose cushion purification supe. 3) 3.5x10^4^ PFU ZIKV positive control. 4) pCMV-prM/E transfection post sucrose cushion purification pt. 5) pCMV-GFP transfection post sucrose cushion purification pt. 1C-E: Sucrose cushion purified Zika VLPs observed using transmission electron microscopy. 1C: VLPs stained with Tungsten. Diameter is indicated. Background protein staining also apparent. 1D: VLP stained with Tungsten. Membrane proteins visible on the surface of VLP are indicated with arrow. Background protein staining apparent. 1E: VLP stained with Uranyl acetate. Membrane proteins visible on the surface of VLP are indicated with an arrow.

The prM and E genes of ZIKV strain H/PF/2013 with nascent signal sequence were cloned into a pCMV expression vector under the control of a cytomegalovirus (CMV) promoter and CMV polyadenylation signal (pCMV-prM/E, Fig 1.). Endotoxin free, transfection grade DNA was prepared using Maxiprep kit (Zymo Research, Irvine, CA). VLPs were expressed by transfecting 90% confluent monolayers of HEK293 cells in a T-75 flasks with 15μg of pCMV-prM/E using Fugene HD (Promega, Madison, WI) transfection reagent according to manufacturer protocol. The 10 ml supernatant was harvested 72hr after transfection, and clarified by centrifugation at 15,000 RCF for 30 min at 4° C. Clarified supernatants were layered onto a 20% sucrose cushion and ultra-centrifuged in a SW-28 rotor at 112,000 RCF for 3.5 hours at 4° C. Pellet (PT) and supernatant (SUP.) fractions at each step were saved for analysis by SDS-PAGE and Western blot. Post sucrose cushion PT were resuspended in Phosphate Buffered Saline (PBS) pH 7.2. Total protein in VLP preparations was quantified by Bradford assay. VLP specific protein was determined by comparing Zika specific bands on SDS-PAGE gels to known concentrations of BSA using ImageJ software.

### Western blot

VLP fractions were boiled in Laemmli sample buffer (BioRad, Hercules, CA, USA) and resolved on a 4–20% SDS-PAGE gel (Biorad) by electrophoresis using a Mini-PROTEAN 3 system (BIO-RAD, CA). Gels were electroblotted onto nitrocellulose membranes using a Turboblot system. Membranes were blocked in 5% (W/V) skim milk and probed with mouse hyper immune ascites fluid primary antibody (1:5000) and goat anti-mouse HRP conjugated secondary antibody (1:5000). Membranes were developed using a solid phase 3,3’,5,5’-tetramethylbenzidine (TMB) substrate system.

### Transmission electron microscopy

Samples were negatively stained for electron microscopy using the drop method. A drop of sample was placed on a PioloformTM (Ted Pella, Inc.) carbon-coated 300 Mesh Cu grid, allowed to adsorb for 30 seconds, and the excess removed with filter paper. Next, a drop of methylamine tungstate or uranyl acetate (Nano-W, Nanoprobes Inc.) was placed on the still wet grid, and the excess removed. The negatively stained sample was allowed to dry, and was documented in a Philips CM120 (Eindhoven, The Netherlands) transmission electron microscope at 80kV. Images were obtained using a SIS MegaView III digital camera (Soft Imaging Systems, Lakewood. Colorado).

### Vaccination and viral challenge

Each of the following animal studies was performed as one biological replicate. For VLP formulations, the indicated dose of sucrose cushion purified VLPs was mixed with 0.2% Imject Alum (Thermo Scientific) according to manufacturer’s protocol. Groups of AG129 mice were injected intramuscularly (IM) with VLPs mixed with alum (n = 5) or PBS mixed with alum (n = 6) at 6 weeks of age, and again at 8 weeks of age. Sub-mandibular blood draws were performed pre boost and pre challenge to collect serum for analysis by neutralization assays and for passive transfer studies.

AG129 mice were challenged with 200 PFU of ZIKV strain H/PF/2013 in 25μl volumes by intradermal (ID) injection into the right hind footpad at 11 weeks of age. BALB/c mice were vaccinated once at 5 weeks of age as above, and challenged at 13 weeks of age with 200 PFU of H/PF/2013 in 50μl by retro orbital injection (IV route).

Following infection, mice were monitored daily for the duration of the study. Mice that were moribund or that lost greater than 20% of starting weight were humanely euthanized. Sub-mandibular blood draws were performed on day two post challenge (PC) and serum collected to measure viremia.

Eight week old AG129 mice were used for passive transfer studies Five naive mice were injected intraperitoneally (IP) with 500μl of pooled serum from VLP vaccinated, diluted serum (1:5 n = 4, 1:10, n = 4), or serum from PBS/alum (n = 5) treated mice. At 12h post transfer, mice were challenged with 20 PFU in 25μl as above.

### Viremia assays

Viremia was determined by TCID_50_ assay. Briefly, serum was serially diluted ten-fold in microtiter plates and added to duplicate wells of Vero cells in 96-well plates, incubated at 37°C for 5 days, then fixed and stained with 10% (W/V) crystal violet in 10% (V/V) formalin. Plates were observed under a light microscope to determine the 50% tissue culture infective doses (TCID_50_s). Serum samples were also tested for viral RNA copies by qRT-PCR. RNA was extracted from 0.02ml of serum using the ZR Viral RNA Kit (Zymo Research, Irvine, CA). Viral RNA was quantified by qRT-PCR using the primers and probe designed by Lanciotti et al [52]. The qRT-PCR was performed using the iTaq Universal Probes One-Step Kit (BioRad, Hercules, CA) on an iCycler instrument (BioRad, Hercules, CA). Primers and probe were used at final concentrations of 500 nM and 250 nM respectively. Cycling conditions were as follows: 50°C for 10 min and 95°C for 2 min, followed by 40 cycles of 95°C for 15 sec and 60°C for 30 sec. Virus concentration was determined by interpolation onto an internal standard curve made up of a 5-point dilution series of in vitro transcribed RNA, with the lowest copies per reaction being 100.

### Neutralization assay

Serum antibody titers were determined by microneutralization assay. Briefly, serum was incubated at 56°C for 30 min to inactivate complement and then serially diluted two-fold in microtiter plates. 200 PFUs of virus were added to each well and incubated at 37°C for 1h. The virus-serum mixture was added to duplicate wells of Vero cells in 96-well plates, incubated at 37°C for 5 days, then fixed and stained with 10% (W/V) crystal violet in 10% (V/V) formalin, then observed under a light microscope. The titer was determined as the serum dilution resulting in the complete neutralization of the virus.

### Plaque reduction neutralization test

Serum samples were serially diluted, mixed with 200 PFU of the ZIKV H/PF/2013 strain and incubated for 1 hr at 37°C. This serum/virus mixture was added to confluent layers of Vero cells in 96 well plates and incubated for 1 hr at 37°C, after which the serum/virus mixture was removed and overlay solution (3% CMC, 1X DMEM, 2% FBS and 1X Anti/Anti) was added. After 48 hrs of infection, the monolayers were fixed with 4% PFA, washed twice with PBS, and then incubated with ZIKV hyperimmune mouse ascitic fluid (1:2000, UTMB) diluted in blocking solution (1X PBS, 0.01% Tween-20 and 5% Milk) and incubated overnight at 4°C. Plates were washed three times with PBS-T and then peroxidase-labeled goat anti-mouse secondary antibody (1:2000) was incubated on monolayers for 2 hours at 37°C. Following incubation, cells were washed a final three times with PBS-T and developed using 3-amino-9-ethylcarbazole (AEC)-peroxidase substrate. The amount of formed foci were counted using an ELISPOT plate reader (ImmunoSPOT-Cellular Technology); quality control was performed to each scanned well to ensure accurate counting. Neutralization percentages (Nx) were calculated per sample/replicate/dilution as follows:

$$Nx=\{100-[100(\frac{A}{Control})$$

Where *A* corresponds to the amount of foci counted in the sample and *Control* is the geometric mean of foci counted from wells treated with cells and virus only. Data of corresponding transformed dilutions (Log(1/Dilution)) against neutralization percentages per sample was plotted and fitted to a sigmoidal dose-response curve to interpolate PRNT_50_ values (GraphPad Prism software).

## Results

### Expression and purification of soluble, Zika VLPs

To generate Zika VLPs (ZIKVLPs), we cloned the prM/E genes with native signal sequence into a pCMV expression vector (pCMV-prM/E) (Fig 1A), transfected HEK293 cells and harvested supernatants (supe.) 3 days post transfection. 78μg total protein was recovered from post sucrose purification of which 21.6 μg was ZIKVLP protein. Western blot analysis of this pCMV-prM/E supe. revealed expression of a ~50kDa size band (Fig 1B, lane 2) that corresponded in size to the predicted size of the Zika virus E gene, and additionally matched positive control Zika virus stocks (Fig 1B, lane 3). To test the hypothesis that expression of Zika prM and E genes spontaneously form extracellular particles, supernatants from pCMV-prM/E and pCMV-GFP (negative control) transfected cells were centrifuged on a sucrose cushion (SC) sufficient for pelleting of flavivirus particles from cell culture proteins [27]. pCMV-prM/E SC purified pellet (pt.) appeared to contain high levels of E protein, indicating that staining was specific to expression of prM and E genes. Purity of ZIKVLP preparations was additionally analyzed by SDS-PAGE of VLP preprations, followed by total protein staining, which revealed only major staining at E protein specific bands (Sup. Fig 1A and 1B). Additionally, western blot analysis using monoclonal antibody specific to ZIKA virus E protein revealed E protein at the expected size (Sup. Fig 2C).

**Fig 2:**
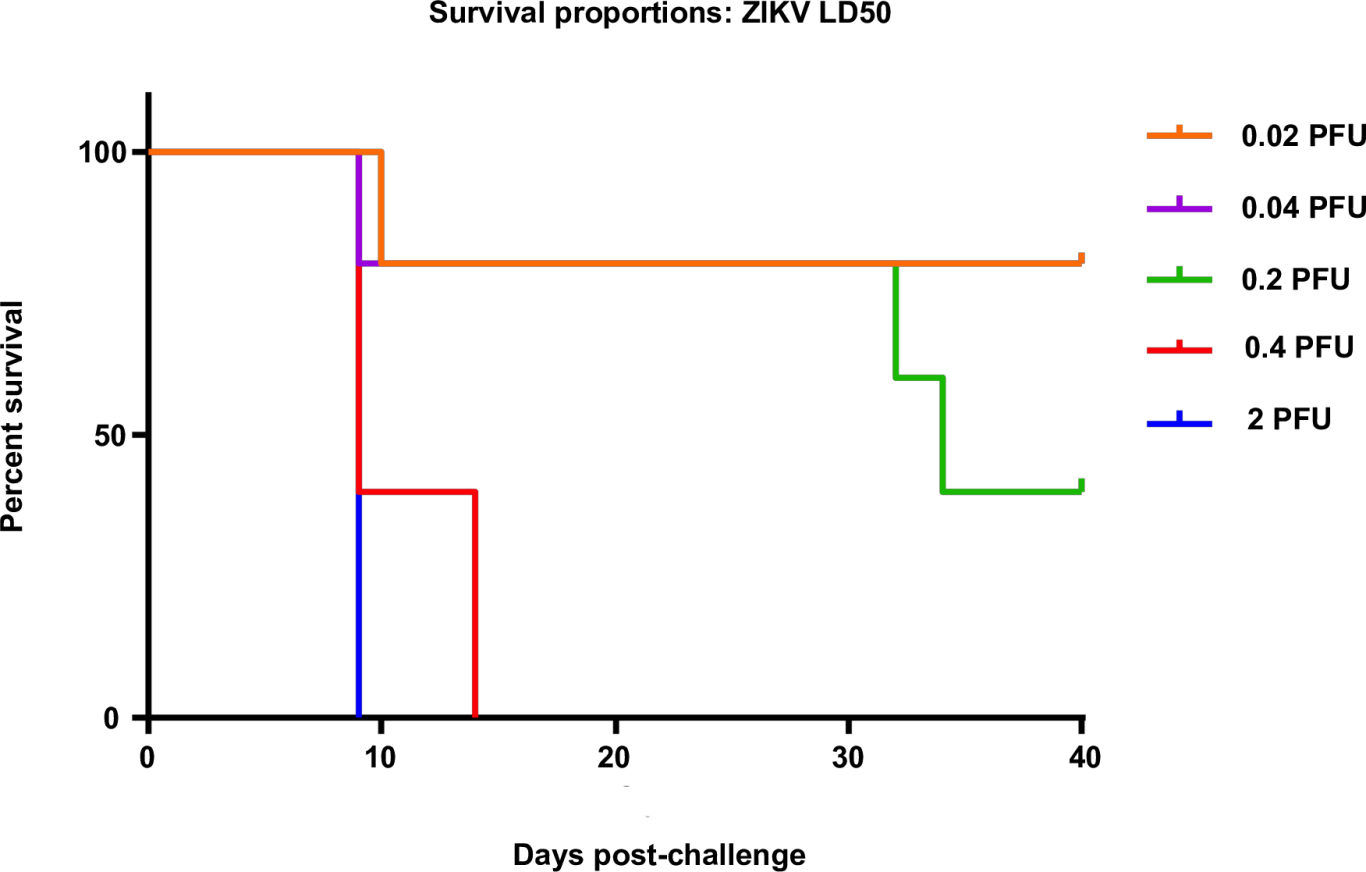
LD50 of ZIKV in AG129 mice. Survival of AG129 after ZIKV over a 14 day period.

To determine if the immune reactive extracellular particles were resembled ZIKV, we performed transmission electron microscopy (TEM) on pCMV-prM/E SC pt. material. TEM revealed structures with similarity to ZIKV and size that ranged from 30-60nm, and an average size of ~50nm (Fig 1C–1E).

### Administration of ZIKVLPs is immunogenic and protects highly ZIKV susceptible α/β/γ interferon deficient (AG129) mice

We first determined the LD_50_ of the H/PF/2013 strain in 12 week-old mixed sex AG129 mice. Groups of mice (n = 5) were infected with 5-fold serial dilutions from 2 PFU to 0.02 PFU of ZIKV and monitored for 4 weeks following the last mortality. All mice infected with 2 or 0.4PFU died within the first week of challenge (Fig 2), while lower doses killed only 1 to 2 mice within the first two weeks. Interestingly, 2 mice infected with 0.2 PFU ZIKV became ill and were euthanized due to weight loss and paralysis 4.5 weeks following challenge. The resultant LD_50_ value in PFUs was calculated to be 0.19 PFU by the Reed-Muench [28] method.

**Fig 3:**
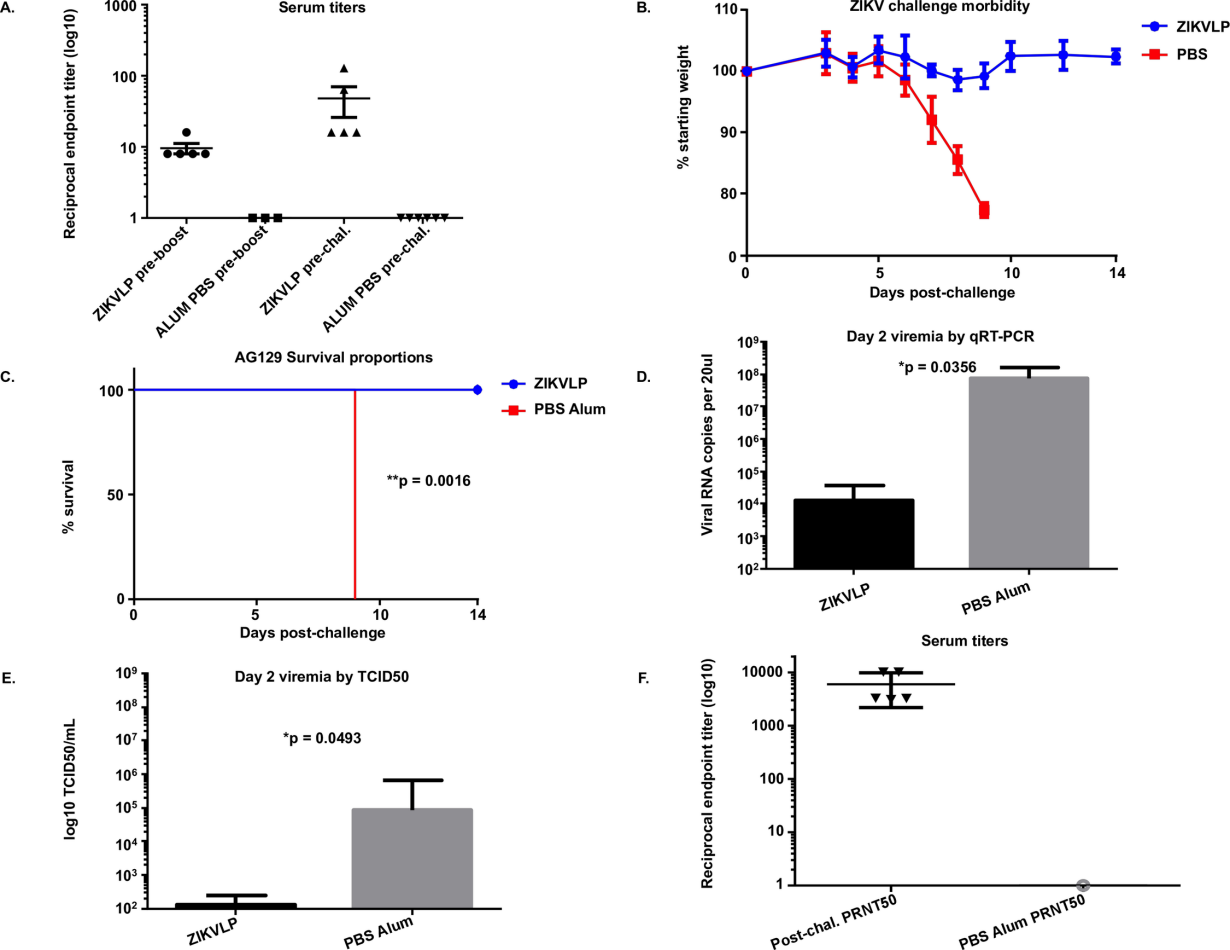
Protection of ZIKVLPs in AG129 mice. 3A: Neutralizing antibody titers (+/- SD) of vaccinated AG129 mice pre boost and pre challenge. 3B: Average weight loss (+/- SD) of AG129 after ID challenge with 200 PFU ZIKV over a 14 day period. 3C: Survival of 11 week old AG129 after ID challenge with 200 PFU ZIKV over a 14 day period. 3D: Viremia (+/- SD) in serum samples from mice two days post challenge by qRT-PCR. Values are total RNA copies per reaction. 3E. Viremia (+/- SD) in serum samples from mice two days post challenge by TCID_50_. 3F: PRNT_50_ values (+/- SD) of serum samples taken from ZIKVLP vaccinated AG129 mice post challenge, and pre challenge serum from PBS/alum mice.

To determine if ZIKVLPs are immunogenic and protective in highly susceptible AG129 mice, groups of mice received a prime and boost of 450ng ZIKVLPs. AG129 mice that received ZIKVLPs developed low levels (GMT = 1:9.2) of neutralizing antibodies (nAbs) at two weeks post administration (Fig 3A), that increased two weeks after boost (GMT = 1:32). Five weeks after primary vaccination, all mice were challenged with 200 PFU (>1000LD_50_s) of ZIKV by the ID route. Mice administered ZIKVLPs maintained weight, while mice that received PBS/alum experienced significant morbidity throughout the challenge period (Fig 3B). All control mice (survival 0/6) died 9 days after ZIKV challenge and had significantly lower survival (p = 0.0016) than mice administered ZIKVLPs (survival 5/5, Fig 3B and 3C). Finally, ZIKVLPs vaccinated mice had significantly lower levels of viremia on day 2 post challenge than control mice detected by qRT-PCR (ZIKVLP = 1.3x10^4^ RNA copies, PBS/alum 9.6x10^7^ RNA copies, p = .0356, Fig 3D) and TCID_50_ assay (ZIKVLP = 1.3x10^2^ TCID_50_s, PBS/alum 2.8x10^5^ TCID_50_s p = .0493, Fig 3E).

### ZIKVLPs elicit plaque reducing neutralizing antibody titers in mice that can be passively transferred to naïve mice

The plaque reduction neutralization test (PRNT) assay is widely considered to be the “gold standard” for characterizing and quantifying circulating levels of anti-dengue and other flaviviral neutralizing antibodies (nAb)[29]. We therefore developed and optimized a PRNT assay for rapidly measuring ZIKV specific neutralizing antibodies. Pooled serum samples collected from mice pre-challenge, as well as individual serum samples collected from mice post-challenge were tested by this PRNT assay. Pre challenge, pooled serum from mice administered ZIKVLPs had a calculated 50% plaque reduction (PRNT_50_) titer of 1:157. The PRNT_50_ titer increased 2 weeks post challenge (GMT = 5122) (Fig 3F).

To test the role of anti-ZIKV antibodies in protection against challenge, groups of mice received ZIKVLP antiserum (pooled pre challenge serum, titer in Fig 3F), undiluted (n = 5), diluted 1:5 (n = 4), or 1:10 (n = 4). As a negative control, mice (n = 5) were transferred serum from mice previously vaccinated with PBS alum.

**Fig 4:**
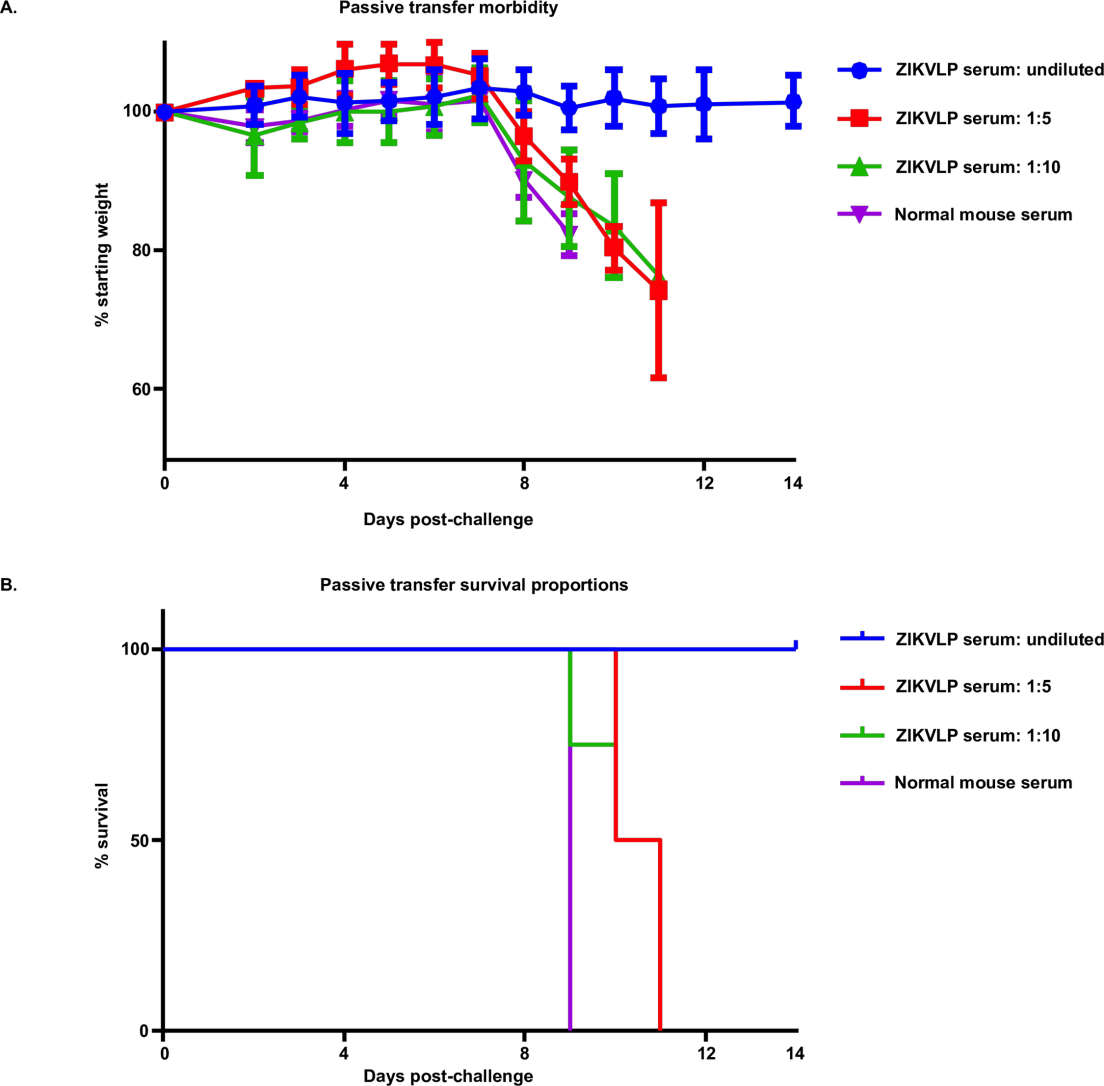
ZIKVLP serum transfer to naïve AG129 mice. 4A: Average weight loss (+/- SD) of 8 week AG129 transferred serum from mice vaccinated with ZIKVLPs after ID challenge with 20 PFU of ZIKV over a 14 day period. 4B: Survival of AG129 after challenge with ZIKV over a 14 day period.

Negative control mice rapidly lost weight starting after day 7 and all died day 9 post challenge (Fig 4A and 4B). Mice that received undiluted serum maintained weight throughout the 14 day period post challenge, and showed no signs of infection. Mice that received diluted anti-ZIKV antibodies were not protected from challenge, although survival and weight loss were slightly extended relative to negative control mice (Fig 4A and 4B).

### A single dose of ZIKVLPs can protect highly susceptible AG129 mice

**Fig 5:**
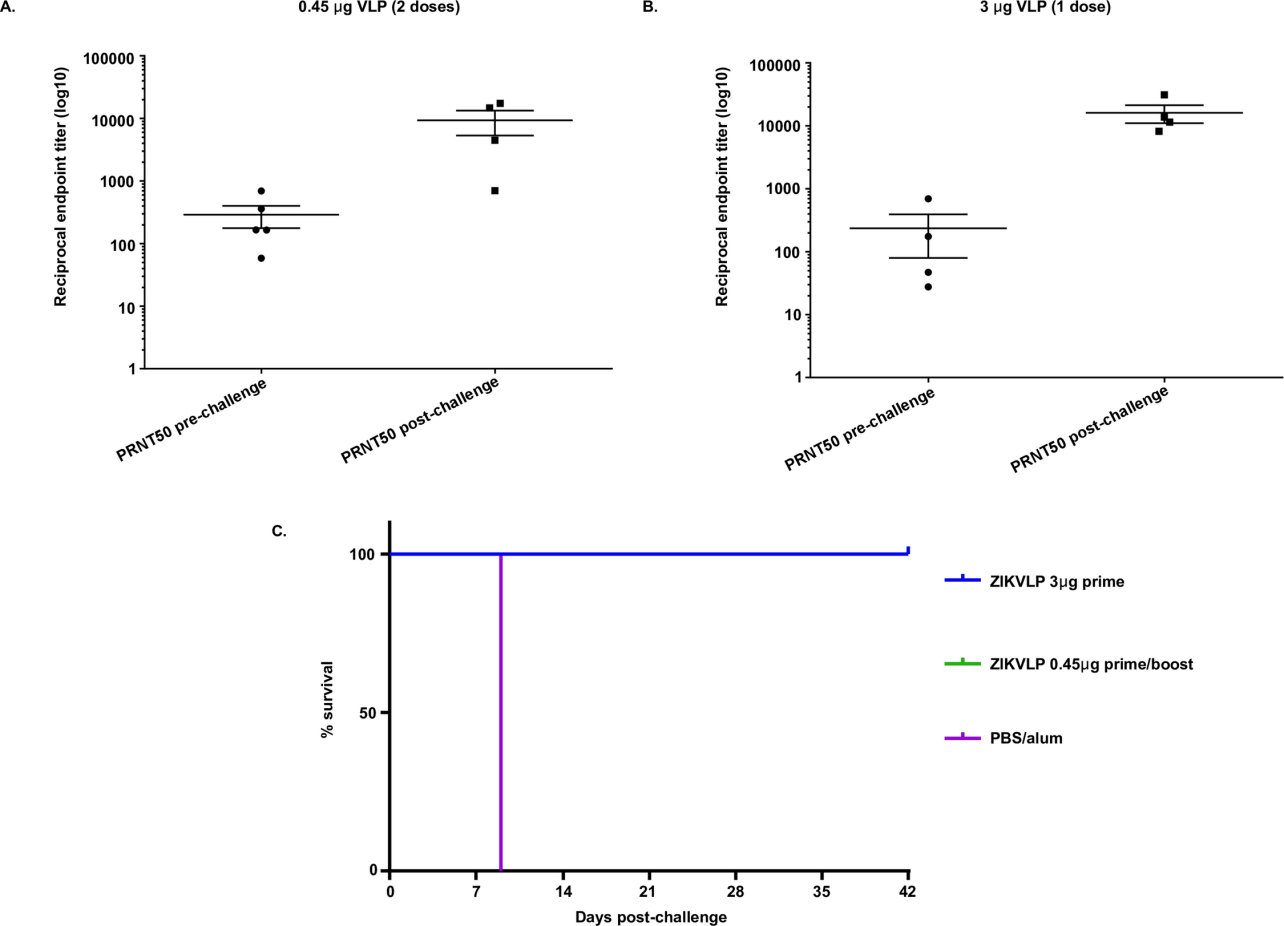
Dose response of ZIKVLPs in AG129 mice. 5AB: PRNT_50_ values (+/- SD) of serum samples taken from AG129 mice administered a prime and boost of 0.45 μg (5A) or a prime only of 3.0 μg (5B) ZIKVLPs pre and post challenge. 5C: Survival of 11 week old AG129 after ID challenge with 200 PFU ZIKV over a 14 day period.

To determine if a single dose could protect AG129 mice, groups of 6-week old AG129 mice were vaccinated with 3μg ZIKVLPs adjuvanted with alum. An additional group of mice (n = 5) was vaccinated with a prime and boost of 0.45μg adjuvanted with alum for comparison. Negative control mice (n = 5) received a prime and boost of PBS/alum. Vaccinated mice developed neutralizing antibodies measured by PRNT assay prior to challenge (Fig 5A). Eight weeks following primary vaccination mice were challenged with 200 PFU (>1000LD_50_s) of ZIKV by the ID route. All mice administered a prime of 3μg or a prime and boost of 0.45μg ZIKVLPs survived throughout the 6 week challenge period (Fig 5C) and maintained weight throughout the challenge period. Pre challenge neutralizing antibody titers in both single (GMT PRNT50 = 288) and double dose (GMT PRNT50 = 235) groups increased significantly (p < .005) in all animals measured at 3 weeks post challenge (Fig 5A and 5B).

### ZIKVLPs protect wildtype BALB/c mice

**Fig 6:**
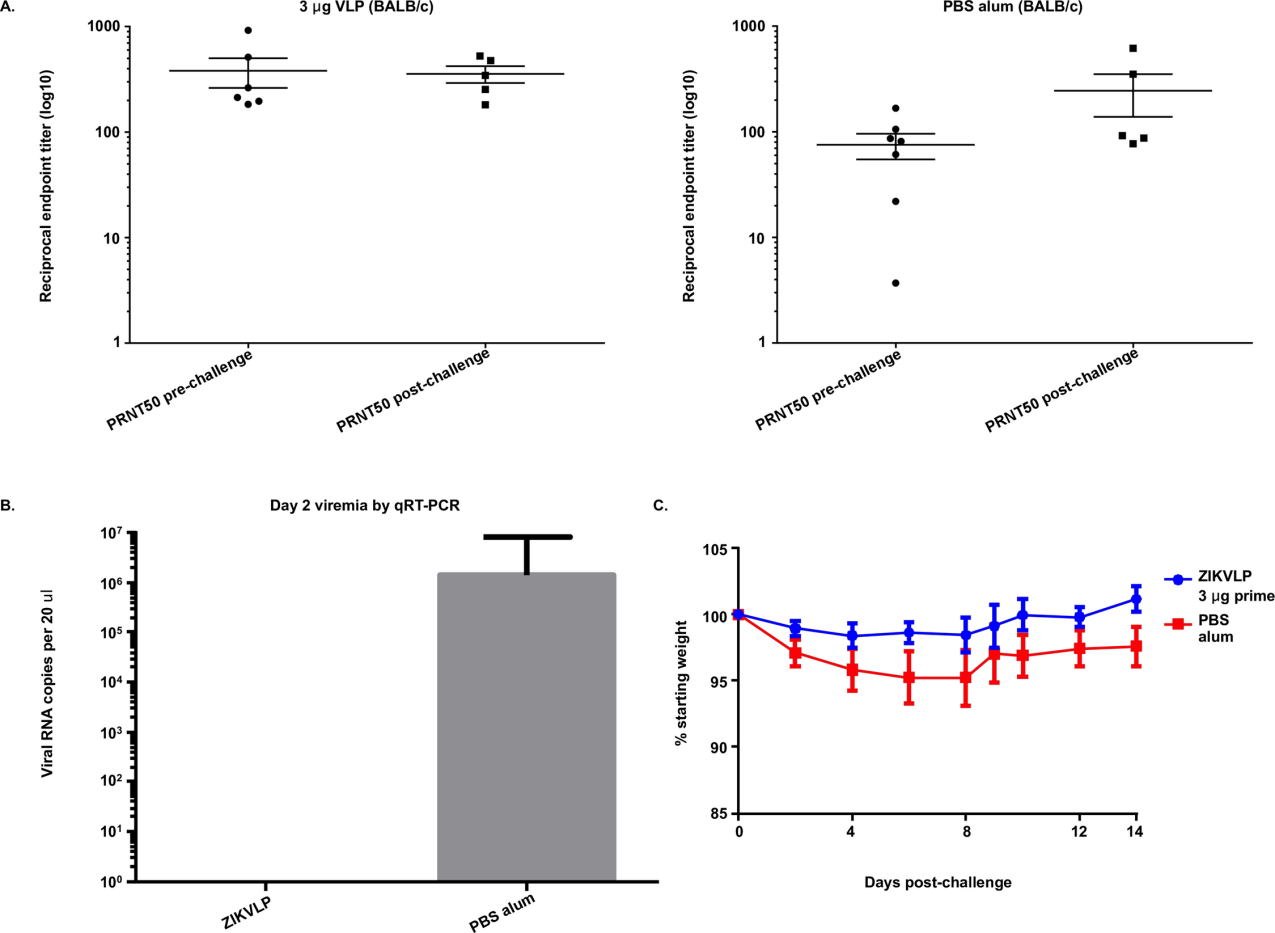
Protection of ZIKVLPs in BALB/c mice. 6A: PRNT_50_ values (+/- SD) of serum samples taken from BALB/c mice administered a prime only of 3.0 μg ZIKVLPs post challenge. 6B: Viremia (+/- SD) in serum samples from mice two days post challenge by qRT-PCR. Values are total RNA copies per reaction. 6C: Average weight loss (+/- SD) of BALB/c mice after ID challenge with 200 PFU ZIKV over a 14 day period.

To determine if ZIKVLPs can protect wildtype BALB/c mice against non-lethal ZIKV challenge, a group (n = 6) was vaccinated with a single dose of 3μg ZIKVLPs adjuvanted with alum. Negative control mice (n = 5) were administered PBS/alum. Eight weeks after vaccination mice were challenged with 200 PFU ZIKV by the IV route. A single dose of ZIKVLPs elicited high titers of neutralizing antibodies (PRNT50 = 381) detected immediately prior to challenge (Fig 6A). Mice vaccinated with ZIKVLPs were completely protected from viremia on day 2 post challenge (Fig 6B), and maintained weight throughout the challenge period (Fig 6C). Negative control animals lost minor amounts of weight beginning at day 2 post challenge, had high levels of viremia and recovered by 2 weeks post challenge. Neutralizing antibodies were undetectable in negative control mice prior to challenge, but increased significantly after challenge (Fig 6A). Antibody titers in vaccinated mice decreased, but were not significantly different than before ZIKV challenge (Fig 6A). Serum from mice prior to challenge (8 weeks post vaccination) was also analyzed by ELISA. ZIKVLP vaccination induced high levels of IgG1, IgG2A/B and low levels of IgM relative to mock vaccinated controls at both 2 and 8 weeks following vaccination.

## Discussion

In February 2016, the World Health Organization declared that the recent clusters of microcephaly and other neurological disorders in Brazil constitute a public health emergency of international concern. Their recommendations included enhanced surveillance and research, as well as aggressive measures to reduce infection with Zika virus, particularly amongst pregnant women and women of childbearing age. As of December 2017, the Zika epidemic has resulted in at least 223,336 confirmed cases worldwide with 3,715 cases of confirmed congenital syndrome associated with ZIKV infection [30]. The trends in Zika and Guillain-Barre syndrome cases should raise major concerns while at least five countries in the Americas have reported sexually transmitted Zika cases, adding to the list of alarming risk factors. ZIKV is now receiving considerable attention due to its rapid spread in the Americas, and its association with microcephaly [31] and Guillain-Barre syndrome [9]. Since the declaration of this public health emergency, several ZIKV vaccine candidates have been evaluated and even tested in animal models. Producing an effective and safe control strategy against the spread of ZIKV and its associated syndromes is still of high priority. In our studies, we designed a ZIKV-virus-like particle (VLP) vaccine, demonstrated expression *in vitro* by western blot and transmission electron microscopy, and tested the protective efficacy and role of antibodies in protection in the AG129 mouse model. This work provides additional support to previous reports on the value of ZIKV-VLP vaccines [19, 32]. In comparison to inactivated vaccine formulations, ZIKVLPs induced significantly higher neutralizing antibody titers that reacted to conformational neutralizing epitopes. Our study differs from previous VLP work on several aspects: 1) evaluation of single vs multiple vaccine doses; 2) role of antibodies on protection using a passive transfer model; and 3) efficacy using both immunocompetent (BALB/c) and susceptible (AG129) mouse models.

Although the transfection and purification procedures for this ZIKVLP have yet to be optimized, we had an overall calculated yield of 2.2 mg/L. Similar expression levels have been reported for other flavivirus VLP expression strategies [33]. Future work will optimize VLP production and purification parameters, which should significantly increase both yield and purity. We are currently developing stably transfected HEK cells that continuously express VLPs, which will allow for scalable production to help meet global demand for a ZIKV vaccine, which is estimated to be 100 million doses a year.

ZIKVLPs, formulated with alum, induced detectable neutralizing antibodies and protected animals against lethal challenge (>400 LD_50_s) with no morbidity or mortality. Pre-challenge GMT neutralizing titers were 1:32, and pooled pre-challenge serum PRNT_50_ titer was 1:157. At a relatively low dose of 450ng, our results indicate that our ZIKVLPs are highly immunogenic. Initial doses (450ng) were chosen based on available antigen. Since 2 doses of 0.45ug did not elicit complete protection in AG129 mice, a single, larger dose (3ug) was chosen to determine if a single dose could provide complete protection in both mouse models. The antibody titers we obtained are consistent with those reported for other highly immunogenic flavivirus VLP vaccines [33, 34]. Previous work has shown a direct correlation between dose of VLPs and neutralizing antibody titers. For ZIKV, questions remain about the quantitative relationship between dose of VLPs and their effect on neutralizing antibody titers and protection from ZIKV challenge *in vivo*.

In our studies, mice were vaccinated with ZIKVLPs and challenged with a homologous strain of ZIKV (H/PF/2013), which raises the question of ZIKVLP specific antibody cross reactivity to heterologous viruses currently circulating in the Americas. Although the H/PF/2013 virus was isolated well before the current outbreak from a patient infected in French Polynesia, there is a high degree of amino acid similarity (~99%) to endemic South American strains of ZIKV [35, 36]. Some experts agree that the high serological cross-reactivity among ZIKV strains would allow for a monovalent vaccine [15]. Nevertheless, care must be taken to empirically determine if antibody responses elicited by ZIKVLPs cross-react and protect against South American strains. Finally, any future ZIKV vaccination programs should incorporate careful surveillance of circulating strains to help suppress immunological escape, and ensure efficacy of vaccines in human populations.

Vaccinated AG129 mice challenged with >1000 LD_50_s had low levels of viremia (1.3x10^2^ TCID_50_s, Fig 3E) detected after challenge. Copies of RNA ZIKV genomes in serum of mice were significantly higher than levels of viremia. However, the disparity between viral genome copies and viremia has been observed for other flaviviruses including dengue [37]. Since AG129 mice are highly susceptible to viral challenge, it is possible that the challenge dose given for the active vaccination study was artificially high. Methods for challenging mice from infected mosquito bite should be developed to most accurately mimic natural infection. The most important criteria for any ZIKV vaccine is its ability to prevent placental and fetal pathology in ZIKV infected pregnant women. Recently developed IFN deficient pregnant mouse models can provide an opportunity to assess if vaccination of pregnant animals can protect the fetus from ZIKV-induced pathology [38]. Although models for ZIKV infection in pregnant non-human primates (NHP) are still being developed, ZIKV vaccines should be tested in NHP translational models which most accurately mimics human immune responses to vaccination.

A VLP vaccine approach against ZIKV has significant advantages over other technologies as it will eliminate concerns of live attenuated vaccines and insufficient inactivation of killed vaccines for pregnant women and other populations at high risk of suffering the devastating effects of ZIKV infections. Production of inactivated vaccines requires high titer growth of infectious virus which may pose a safety concern for workers. Additionally, the production of both attenuated and inactivated ZIKV vaccines is limited to “batch” production, whereas flavivirus VLPs can continuously expressed from stable cell lines. In recent years, recombinant virus-like particle (VLP)-based vaccine strategies have been frequently used for vaccine design. VLPs are known to be highly immunogenic and elicit higher titer neutralizing antibody responses than subunit vaccines based on individual proteins [39].

The role of neutralizing antibodies in protecting against ZIKV was demonstrated by antibody passive transfer studies as naive AG129 mice receiving pooled serum from VLP vaccinated animals were fully protected. These results are consistent with previous findings that indicate the important role of antibodies in protecting against many insect-borne flaviviruses, such as Japanese encephalitis, west Nile virus, and tick borne encephalitis [40–42], even at low levels of circulating antibodies. In this study, we observed full protection when animals received undiluted serum (PRNT_50_ 1:157), with no weight loss or other clinical signs observed.

At the time of this passive transfer study, we had not finished generated data regarding low doses of ZIKV and mortality past 14 days. However, according to our complete studies [43], the challenge dose (20 PFU) should have resulted in 100% mortality by day 9, therefore, all mice challenged and alive through day 14 indicate protection against ZIKV infection. While these studies highlight the importance of serum antibodies in ZIKV protection, there are still many important questions related to ZIKV immunology. What is the minimum antibody titer needed for protection, do ZIKVLPs elicit CD8+ responses and are these responses involved in protection, and what is the overall role of cellular immunity in protection? It is also important to determine if anti-ZIKV antibodies, particularly those elicited by ZIKVLPs, play any role in dengue protection or disease enhancement. Of note, vaccination with VLPs elicited low levels of IgM relative to IgG1/2 following vaccination, which may be important for compatibility with IgM detection kits for use during pregnancy. In this study we used AG129 IFN receptor-deficient mice. These mouse models are commonly used for the evaluation of arboviral vaccines, including dengue, chikungunya and yellow fever virus [44–46]. We recently documented the suitability of mice deficient in IFN-α/β and -γ receptors as an animal model for ZIKV, as they are highly susceptible to ZIKV infection and disease, developing rapid viremic dissemination in visceral organs and brain and dying 7–8 days post-infection [43], and evaluated doses as low as 1 PFU. In our current studies, we observed consistent lethality at doses below 1 PFU, indicating that there are viral subpopulations refractory for the formation of CPE in cell culture, but still capable of establishing a lethal infection in highly susceptible mice. It is of great interest is that at a very low dose (0.2 PFU) two of five mice became ill more than 1 month after infection, as infection with ZIKV typically produces rapid lethality in AG129 mice.

Our current studies challenged mice with 200 PFU at 11 weeks of age. All control mice lost 20% weight, were moribund, and succumbed to by challenge by day 9. ZIKV challenge therefore appears to be completely lethal in both juvenile and adult AG129 mice. The AG129 mouse model exhibits an intact adaptive immune system, despite the lack of an IFN response, and it has been used extensively to evaluate vaccines and antivirals for DENV [47–50]. In our studies WT BALB/c mice did not succumb to infection with ZIKV consistent with previous studies where BALB/c mice were experimentally inoculated with 200 PFU of ZIKV [51]. Mice also developed high levels of viremia following IV inoculation. A single dose of VLPs prevented detection of viral RNA copies in serum of vaccinated mice at 2 days post infection–when viremia levels typically peak in the BALB/c model. It is possible that viral replication was completely inhibited, as there was no “boost” response in neutralizing antibodies observed following challenge. Finally, in repeat AG129, and BALB/c mice mouse studies, animals were protected from ZIKV challenge 8 weeks after vaccination. ZIKVLP therefore appear to elicit a potent “memory” response.

In the present study, we used aluminum hydroxide (commonly known as alum) as the adjuvant for ZIKVLP preparations. Since its first use in 1932, vaccines containing aluminum-based adjuvants have been successfully administered in humans demonstrating excellent safety. We intend to examine a variety of adjuvant formulations and optimize their immunogenic potential for our ZIKVLP candidates with a major focus on those that facilitate antigen dose sparing, enhanced immunogenicity, and broadened pathogen protection.

In summary, a vaccine against ZIKV is currently unavailable, nor is there any specific prophylactic treatment. Here we report the initial development of a VLP based Zika vaccine that elicits protective antibodies in mice, and is safe, suitable for scalable production, and highly immunogenic. Fast-tracking development of this ZIKV vaccine is a public health priority and is crucial for restoring confidence and security to people who wish to have children or reside in, or visit areas in which ZIKV is endemic.

## Supporting information

S1 Fig Further western blot analysis of ZIKVLPs A: Coomassie Blue staining of three different batches of ZIKVLPs. Lanes are, 1) Bio-rad precision plus kaleidoscope protein standards. 2–4): ZIKVLP post purification preps 1–3, respectively. B: Western blot analysis of Zika virus like particles using ZIKV+ acetes. Lanes are, 1–3) ZIKVLP post purification preps 1–3, respectively. 4) Bio-rad precision plus kaleidoscope protein standards. 2–4). C: Western blot analysis of Zika virus like particles using ZV-2 monoclonal antibody (BEI NR-50414). Lanes are, 1) Bio-rad precision plus kaleidoscope protein standards. 2) 3.5x10^4^ PFU ZIKV positive control 3) ZIKVLP post purification.
(TIF)

S2 Fig ELISA analysis of IgG1, IgG2ab and IgM in serum collected from mice 2 and 8 weeks post vaccination with ZIKVLPs or PBS alum (8 weeks). Symbols indicate mean OD; error bars indicate standard deviation.
(TIF)
