# Supplementary figures and images for: Zika virus like particles elicit protective antibodies in mice
Source: PLoS Negl Trop Dis. 2018 Feb 5;12(2):e0006210. doi: 10.1371/journal.pntd.0006210 (PMC5814096; doi:10.1371/journal.pntd.0006210)

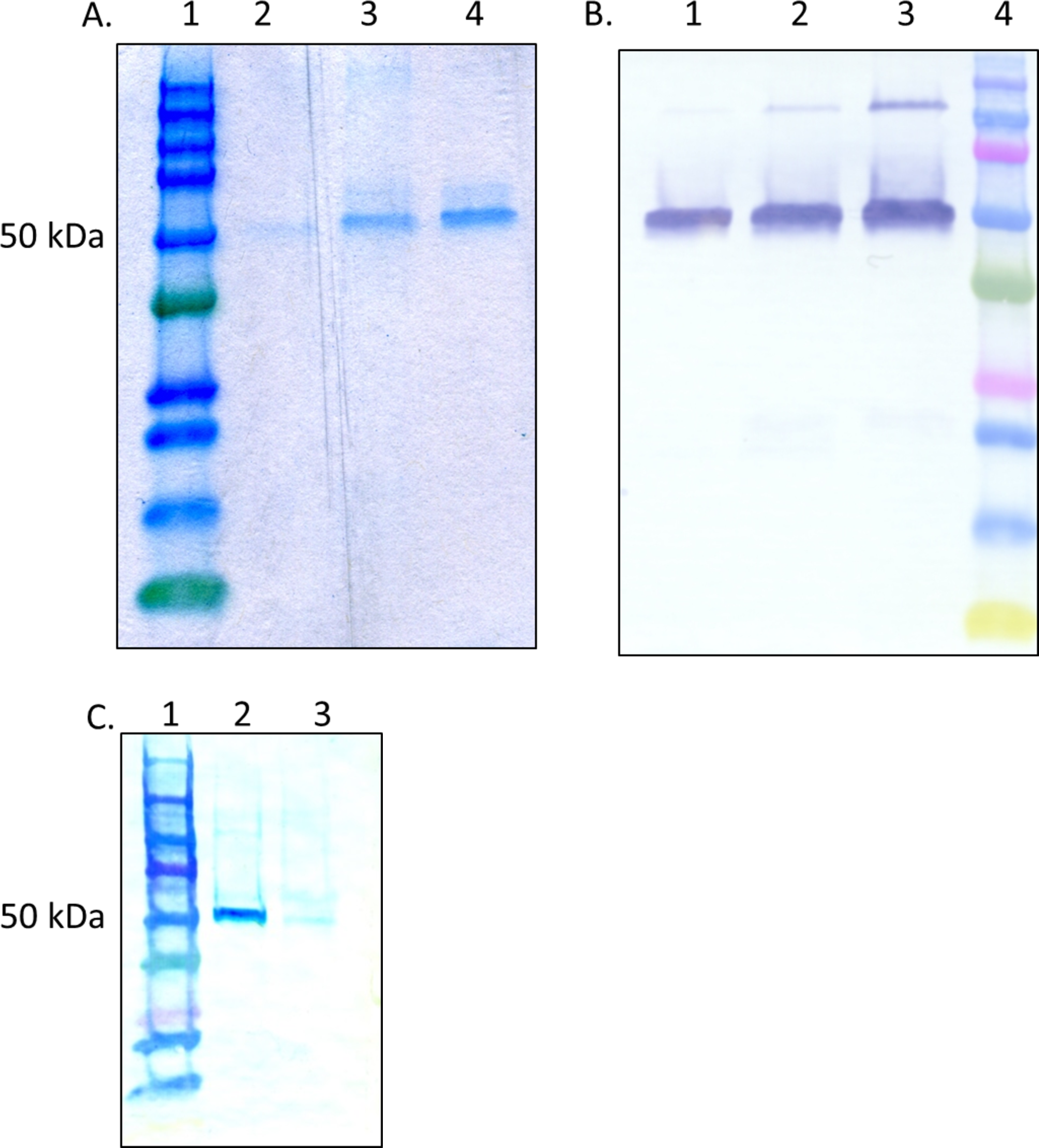

Supplement: S1 Fig — Further western blot analysis of ZIKVLPs A: Coomassie Blue staining of three different batches of ZIKVLPs. Lanes are, 1) Bio-rad precision plus kaleidoscope protein standards. 2–4): ZIKVLP post purification preps 1–3, respectively. B: Western blot analysis of Zika virus like particles using ZIKV+ acetes. Lanes are, 1–3) ZIKVLP post purification preps 1–3, respectively. 4) Bio-rad precision plus kaleidoscope protein standards. 2–4). C: Western blot analysis of Zika virus like particles using ZV-2 monoclonal antibody (BEI NR-50414). Lanes are, 1) Bio-rad precision plus kaleidoscope protein standards. 2) 3.5x10^4^ PFU ZIKV positive control 3) ZIKVLP post purification. (TIF) [file pntd.0006210.s001.tif]

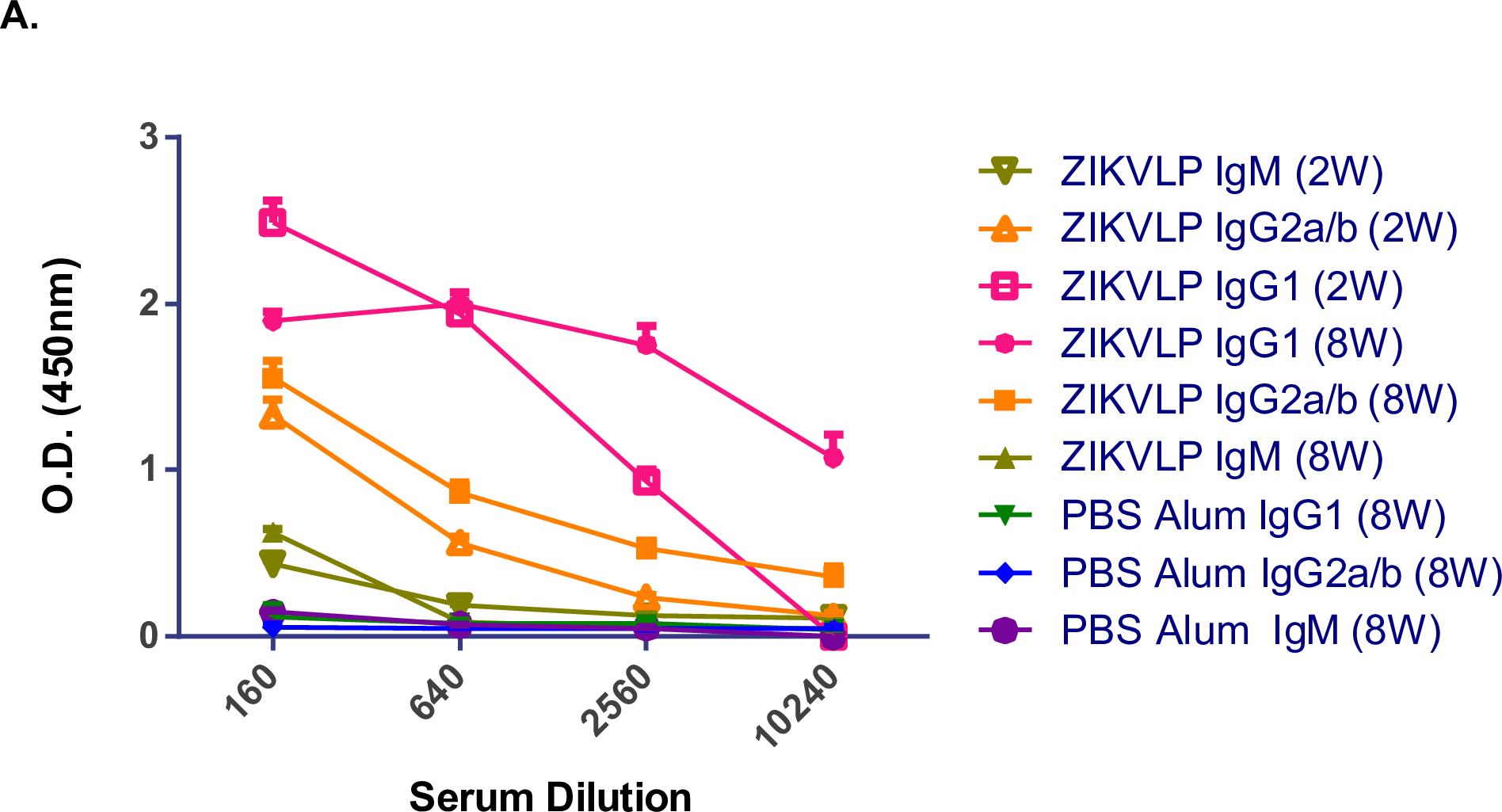

Supplement: S2 Fig — Symbols indicate mean OD; error bars indicate standard deviation. (TIF) [file pntd.0006210.s002.tif]
